# BEYOND THE ENDOTHELIUM: THE ROLE OF MURAL CELLS IN VASCULAR BIOLOGY: *In vitro* systems to study endothelial/pericyte cell interactions

**DOI:** 10.1530/VB-22-0021

**Published:** 2023-02-06

**Authors:** Emily Warren, Sharon Gerecht

**Affiliations:** 1 Department of Biomedical Engineering, Duke University, Durham, North Carolina, USA

**Keywords:** angiogenesis, tissue engineering, microfluidics, microvasculature, vasculogenesis

## Abstract

The vasculature is crucial for tissue development and survival, and the stability of blood vessels to perform these functions relies on the interplay between endothelial cells (ECs) and mural cells. Pericytes are a subtype of mural cells found in the microvasculature that extend their processes to wrap around the endothelial monolayer. Pericytes are recruited during vessel growth through the excretion of soluble factors from ECs where they stabilize angiogenic sprouts and induce maturation of the resident cells. Alterations in these interactions between ECs and pericytes are associated with aberrant vessel growth and disrupted vasculature function characteristic of numerous diseases. Therefore, deeper understanding of the cross-talk between these cell types has numerous implications for understanding morphogenesis and elucidating disease mechanisms. In this review, we highlight recent advances and current trends studying the interactions between ECs and pericytes *in vitro*. We begin by analyzing three-dimensional hydrogel platforms that mimic the tissue extracellular matrix to investigate signaling pathways and altered vascular function in disease-specific cells. We next examine how microfluidic vasculature-on-a-chip platforms have elucidated the interplay of these vascular cells during angiogenesis and vascular network formation under controlled physiochemical cues and interstitial flow. Additionally, studies have utilized microvessels to measure the effect of shear stress on barrier function through the control of luminal flow and the impact of inflammation on these vascular cell interactions. Finally, we briefly highlight self-assembling human blood vessel organoids, an emerging high-throughput platform to study ECs and pericyte interactions.

## Introduction

Understanding the mechanisms governing vascular morphogenesis to efficiently vascularize engineered tissues continues to be a major roadblock in tissue engineering. A fundamental challenge in engineering the complexity of microvascular systems is incorporating multiple cell types in a physiologically relevant environment and spatial arrangement. Interactions between endothelial and mural cells are critical for forming and stabilizing the vasculature. Blood vessels are lined with a monolayer of endothelial cells (ECs) surrounded by mural cells communicating through signaling pathways and direct cell–cell contact to regulate vessel function and development. Mural cells can be subdivided into two cell types: vascular smooth muscle cells (vSMCs) and pericytes, as characterized by differences in cell morphology, location, and density. vSMCs are most commonly found in larger vessels such as arteries and veins, whereas pericytes are located in the microvasculature and capillaries. Although there is no single marker specific to pericytes, several surface markers are commonly used to identify pericytes, specifically platelet-derived growth factor receptor (PDGFR)-β, neural/glial antigen 2 (NG2), and CD13, as well as cytoskeletal proteins such as α-smooth muscle actin (α-SMA), desmin, and calponin (1, 2, 3, 4). Pericytes play a crucial role in blood vessel development, where ECs recruit them through factors such as PDGF-BB to stabilize angiogenic sprouts and induce the differentiation of ECs (5). Disruption of pericyte–EC interactions can lead to altered vasculature function and is associated with numerous disease pathologies such as blood–brain barrier (BBB) dysfunction (6), diabetic retinopathy (7), and tumor angiogenesis (8). Thus, understanding pericyte–EC interactions has numerous implications for understanding vascular development and disease.

Previous *in vivo* studies have informed signaling pathways between ECs and pericytes (9), such as the PDGF-BB/PDGFR-β axis directed from ECs to pericytes (10), the angiopoietin–Tie axis directed from pericytes to ECs (11), and tissue-specific signaling pathways, such as EC–pericyte signaling in the brain (12). Additionally, *in vivo* studies have demonstrated the importance of direct EC–pericyte contact for cell–cell communication through physical interactions, including gap junctions, such as connexin 43 (13), and adherens junctions, specifically N-cadherin (14). However, constructing physiologically relevant *in vitro* systems that successfully incorporate both cell types and the complexity of their interactions continues to be a challenge. *In vitro* culture systems provide numerous advantages, such as tunability of the cellular microenvironment through engineered materials to mimic the extracellular matrix (ECM) and isolate the role of specific matrix properties on vascular function (15, 16, 17). Additionally, using hydrogels to create three-dimensional (3D) models allows for the visualization of vascular network formation and angiogenesis while recapitulating the 3D spatial arrangement observed *in vivo* compared to 2D models. Various cell types and sources can also be incorporated to improve model specificity. Pericytes can be isolated from vascularized tissues such as the brain (18), retina (19), and lungs (20). Pericytes derived from stem cell populations, such as human-induced pluripotent stem cells (iPSCs), are also a desirable cell source as they can be used to create patient-specific models with higher clinical relevance (1, 2).

Engineering vasculature in 3D *in vitro* models with both ECs and pericytes is a significant step toward vascularizing engineered tissues. These platforms also provide the opportunity to elucidate vascular disease progression and serve as drug screening platforms to advance clinical translation. Yet, current models lack the complexity observed *in vivo*, such as necessary biomechanical cues or inflammatory key players such as macrophages. Here, we review recent advances in 3D *in vitro* models to study EC–pericyte interactions in order of increasing complexity and highlight the need for more physiologically relevant constructs of smaller vessels on the scale of the microvasculature.

## Hydrogels

Hydrogels are biomaterials composed of natural or synthetic polymers that aim to recapitulate the ECM of soft tissues and the various biochemical and biophysical cues cells experience *in vivo* that guide cellular processes. Hydrogels are a desirable tool to study EC–pericyte interactions *in vitro* as they provide a physiologically relevant 3D co-culture platform that supports cell proliferation, migration, angiogenesis, and vascular network formation. Here, we review *in vitro* systems composed of 3D hydrogels, primarily collagen-I, Matrigel, or polyethylene glycol (PEG), to inform signaling pathways and pathological interactions between ECs and pericytes.

### Collagen-I hydrogels

Collagen-I hydrogels are a natural biomaterial commonly used to study vascular processes *in vitro*. Collagen-I is an abundant protein in ECM that can self-assemble into a hydrogel when subjected to physiological conditions (21). These natural hydrogels are favorable as *in vitro* cell culture platforms due to their biocompatibility and similarity to ECM, including their fibrous structure, the diffusivity of small molecules, and stiffness reminiscent of soft tissues (21). Previous *in vitro* studies have cultured ECs and pericytes in collagen-I hydrogels to elucidate their interactions under certain conditions. Kemp *et al*. (2020) (22) identified EC-derived growth factors that promote pericyte recruitment, primarily PDGF-BB, PDGF-DD, and endothelin-1, by culturing pericytes along the surface of collagen-I gels (2.5 mg/mL) and measuring their invasion response to each growth factor. The study further confirmed the role of these growth factors by co-culturing ECs and pericytes in collagen-I gels and blocking these growth factors or their receptors on pericytes to confirm decreased capillary network co-assembly. Similarly, Bowers *et al*. (2020) (23) co-cultured ECs and pericytes in collagen-I gels supplemented with stem cell factor (SCF), interleukin (IL)-3, stromal-derived factor (SDF)-1α, fibroblast growth factor (FGF)-2, and insulin to study the effect of priming ECs with vascular endothelial growth factor (VEGF) on EC–pericyte capillary network co-assembly. The studies demonstrated that pretreating ECs with VEGF for 8 h led to an upregulation of PDGF-BB, PDGF-DD, and heparin-binding epidermal growth factor, which have been shown to play a role in pericyte proliferation and recruitment (24). These results were consistent with the observed increase in pericyte recruitment to the EC-lined tubes using the VEGF-primed ECs.

Disease-specific cell types can also be incorporated into collagen-I hydrogel co-culture systems to study pathological alterations in EC–pericyte interactions. A severe genetic risk factor for Alzheimer’s disease (AD) is the ε4 allele of the *APOE* (APOE4) gene that encodes for apolipoprotein E4, and its effect on vascular function is widely unknown. Yamazaki *et al*. (2020) (25) used pericytes isolated from apoE3- and apoE4-targeted replacement mice (apoE3-PCs and apoE4-PCs) to study how apoE isoforms in pericytes alter EC phenotype through the use of collagen-I hydrogels. ECs and apoE3-PCs or apoE4-PCs were co-cultured in collagen-I hydrogels and allowed to form vascular networks. They demonstrated that apoE4-PCs resulted in decreased network formation and shorter branch length than apoE3-PCs, concluding that apoE4 pericytes are inferior at supporting ECs in tube formation. The results of this study inform the potential role of apoE4, pericytes, and altered EC–pericyte interactions in AD and demonstrate the role of collagen-I hydrogels as a useful *in vitro* platform to elucidate pathological EC–pericyte interactions. While this study provides insight into how AD-related mutations alter vasculogenic capacity, AD is a degenerative disease; therefore, a platform to study the deterioration of established vessels in this disease context should be explored as next steps to advance the relevance of these platforms.

### Matrigel

In the microvasculature, pericytes are embedded within the basement membrane (BM) surrounding the EC monolayer (3). A hydrogel known as Matrigel can be formed from BM extracts, including BM proteins laminin-I and collagen IV, and has been used in previous studies as a platform to study ECs and pericytes (26). Mannino *et al*. (2020) (19) cultured human adipose-derived mesenchymal stem cells (hASCs) in a specific pericyte medium to derive pericyte-like cells (PM-ASCs) and tested their interactions with ECs co-cultured in Matrigel. The expression of α-SMA and NG2 confirmed differentiation into pericyte-like cells. Using human retinal ECs, they demonstrated that the hASCs cultured in pericyte medium localized circumferentially around the tubular structure formed by the ECs, similar to human retinal pericytes. Blanchard *et al*. (2020) (27) developed a BBB model by differentiating isogenic APOE3/3 and APOE4/4 iPSCs into brain ECs, pericyte-like cells, and astrocytes and encapsulating them in Matrigel to form vascular networks and study the effect of the *APOE* genotype on amyloid accumulation, a common pathological mechanism in AD (28). Here, differentiation of the iPSCs into pericyte-like cells was confirmed by the upregulation of pericyte-associated genes and lack of markers for fibroblasts and mesenchymal cells. The study demonstrated that the presence of the pericyte-like cells was required for the amyloid beta accumulation and that the APOE4 cell line resulted in higher accumulation than APOE3, informing potential therapeutic targets. It is worth noting that Matrigel contains numerous active growth factors, such as transforming growth factor β (TGF-β) and PDGF, which can influence EC and pericyte behavior (29). If a more matrix-based platform is desired, growth factor-reduced Matrigel is an alternative that can reduce potential confounding variables from the growth factor activity (30).

### Engineered hydrogels (PEG)

Hydrogels can also be engineered to mimic specific microenvironmental cues, such as composition, stiffness, viscoelasticity, and oxygen, to study how altered matrix properties affect cellular processes in certain pathologies and stimuli. However, using engineered hydrogels to study EC–pericyte interactions has yet to be widely explored. Previous studies have used PEG hydrogels, a highly tunable synthetic polymer, as an engineered co-culture hydrogel platform. Matta *et al*. (2021) (31) engineered a porous and fibrillar 3D PEG hydrogel to recapitulate the stiffness, porosity, and composition of brain tissue to study how signaling from the co-culture of EC and pericytes affects neural stem cell (NSC) clustering and migration. They observed that pericytes do not play a major role in the migration, clustering, or phenotypic switch of NSCs when co-cultured with ECs. Roudsari *et al*. (2016) (32) studied the role of ECs and pericytes in tumor angiogenesis and cancer cell migration by co-culturing human umbilical vein endothelial cells (HUVECs), human vascular pericytes, and lung adenocarcinoma cancer cells (344SQ) in a two-layer PEG hydrogel. Here, vascular cells were encapsulated in one layer, and cancer cells were encapsulated in the other. Clusters of the 344SQ cells formed at the interface of the hydrogel layers, and morphological changes in the cancer cells occurred close to the vascular cells. The secretion of TGF-β1 from the vascular cells increased the secretion of VEGF, PDGF-BB, and FGF-2 from the 344SQ cells, indicating a cross-talk between ECs, pericytes, and cancer cells. These studies provide a preview into the opportunities of using engineered hydrogels with tailored matrix properties to better inform the counterplay between ECs, pericytes, and the surrounding ECM.

## Microfluidic vasculature-on-a-chip platforms

**Figure 1:**
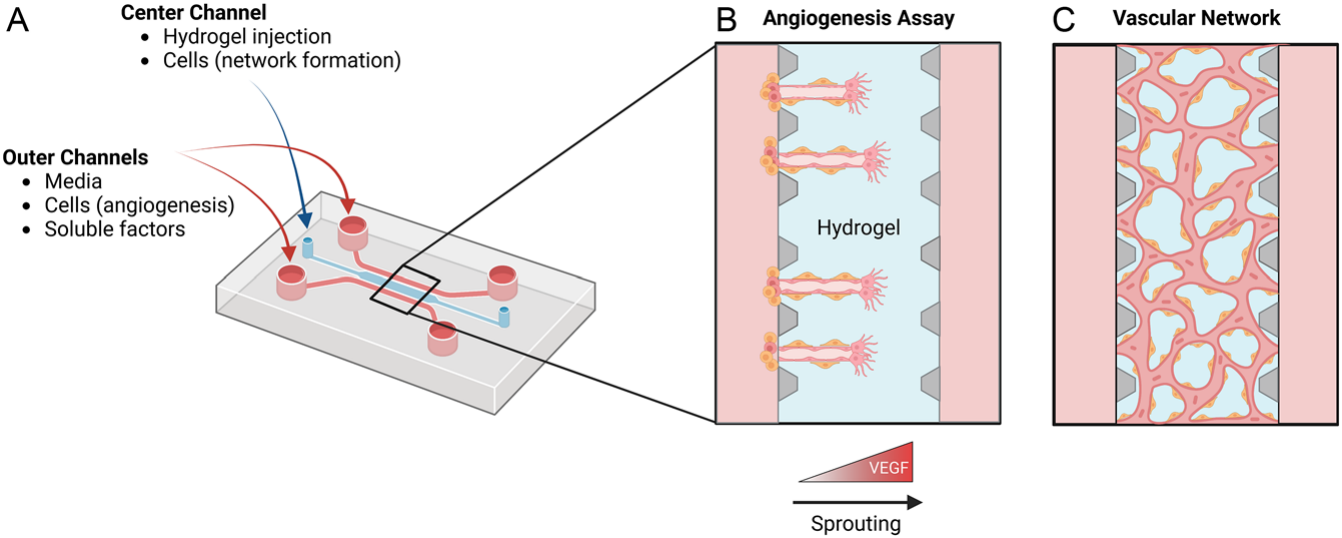
Schematic illustration of microfluidic vasculature-on-a-chip platform. (A) Accessible ports to the microfluidic channels allow for various seeding configurations, media exchange, and the addition of soluble factors. (B) Seeding ECs and pericytes in the outer channels with the addition of a VEGF gradient allows for the visualization of angiogenesis. (C) Vascular networks form within the center channel when ECs and pericytes are suspended within the hydrogel. Created with BioRender.com

Over the years, microfluidics have become an increasingly popular tool to study microvasculature processes *in vitro,* including angiogenesis, vascular network formation, and BBB function (33). These vasculature-on-a-chip platforms combine biophysical approaches to recapitulate the 3D cellular microenvironment *in vivo* that improve upon hydrogels alone, such as advanced cell seeding configurations, chemokine gradients, and mechanical stimuli. They are often designed with a center hydrogel chamber surrounded by two media channels connected to inlet ports where cell seeding, media exchange, and the addition of soluble factors can be achieved (Fig. 1A) (34). These devices are frequently fabricated using polydimethylsiloxane (PDMS), a desirable material due to its ease of use, biocompatibility, and gas permeability (35). The upright posts along the center chamber create surface tension during hydrogel injection to contain the gel solution within the posts and create an accessible gel surface alongside the media channels after polymerization (34). To study angiogenesis, ECs are seeded along the surface of the hydrogel, and a chemokine gradient is added across the media channels to induce invasion and sprouting through the gel (Fig. 1B). To create microvascular networks, ECs are mixed within the gel solution before injection, where they self-assemble into connected capillary-like networks after gel polymerization (Fig. 1C). Multiple cell types, such as pericytes, can be incorporated to increase model complexity and study the interactions between cell types. Here, we review recent vasculature-on-a-chip models used to study various microvasculature processes containing both ECs and pericytes.

Microfluidic models are valuable platforms to study angiogenic responses due to the ability to create directed gradients of soluble factors, such as VEGF, across the hydrogel to visualize invasion and sprouting. Bai *et al*. (2021) (36) used the three-channel microfluidic device to demonstrate how this platform can elucidate the role of pericytes in angiogenesis and study differences in angiogenic response from different strains of brain ECs and pericytes. Specifically, ECs and pericytes were isolated from the brains of two mouse strains classified as either a ‘high’ or ‘low’ angiogenic strain. The ECs and pericytes were co-cultured along the surface of a 3D collagen-I gel using one media channel, and VEGF (25 ng/mL) was added to the opposite media channel to create a gradient across the gel and induce angiogenesis. The physiological process of angiogenesis was observed where vessel sprouts invaded the collagen-I gel, and pericytes migrated along the sprouts formed by ECs. By comparing EC and pericyte co-cultures to ECs alone, pericytes were shown to stabilize the lumen structures formed by ECs, prevent vessel leakage, and play a role in EC proliferation and migration. Additionally, ECs from the ‘high’ angiogenic mouse strain demonstrated longer sprouts and larger vessel diameters than the ECs from the ‘low’ angiogenic mouse strain. Using a similar microfluidic platform setup, Uwamori *et al*. (2019) (37) used a three-channel device to demonstrate differences in the angiogenic responses of organ-specific ECs co-cultured with pericytes. Along the surface of a fibrin gel formed in the center channel, brain microvascular ECs (BMECs) or HUVECs were co-cultured with mesenchymal stem cells (MSCs) that differentiated into pericyte-like cells. The cells invaded the fibrin gel to form angiogenic sprouts where the pericyte-like cells surrounded the ECs. The EC types did not demonstrate differences in pericyte coverage, but differences in the microvascular sprouts were observed, highlighting the need to consider organ-specific cell types when designing cell culture models. Different variations of the three-channel device have also been used. Yamamoto *et al*. (2019) (38) similarly co-cultured HUVECs and pericyte-like cells derived from MSCs alongside the surface of a collagen-I hydrogel in a unique device design. A challenge in co-culturing ECs and pericytes is optimizing the ratio of ECs to pericytes to achieve physiological lumen diameters similar to that of capillaries (<10 μm) and increase lumen stability. Testing HUVEC:MSC ratios of 1:1, 2:1, 5:1, and 10:1 and HUVEC monocultures demonstrated that the 1:1 ratio resulted in capillary-like lumen diameters that remained stable for 21 days. Deposition of BM proteins, laminin and collagen-IV, was confirmed in the 1:1 co-culture, whereas both proteins were not observed in the monocultures. These results further demonstrate the role of EC–pericyte interactions in BM formation, which is important for vessel stabilization.

Microfluidic devices have gained significant popularity in modeling the BBB due to the ability to simultaneously co-culture ECs, pericytes, and astrocytes with advanced cell–cell and cell–matrix interactions. A challenge in developing efficient drugs and therapeutics for brain and neurovascular disease is understanding their transport across the BBB in pathological conditions. Therefore, *in vitro* platforms that recapitulate the BBB microvasculature and effectively model its permeability can be useful in drug development. Campisi *et al*. (2018) (39) used the three-channel device design to create microvascular networks of the BBB by suspending iPSC-derived ECs, human primary brain pericytes, and human primary astrocytes within a fibrin gel. To further mimic the complexity of the BBB, a monolayer of iPSC–ECs was cultured on both sides of the networks using the two outer channels of the device. This novel 3D BBB model formed stable, perfusable vascular networks with physiological diameters and comparable permeabilities. Notably, the results of this study not only supported the dynamic role of pericytes in assisting the formation of stable networks in the BBB but also suggested the role of pericytes in differentiating the iPSC–ECs into brain-specific ECs. As shown in the aforementioned studies, three-channel devices are an established model to study interactions between multiple cell types and the ECM during vascular development. However, five-channel devices can add complexity to these models by utilizing an additional channel for the culture of proangiogenic factor-secreting cells. Fibroblasts, specifically lung fibroblasts, can be cultured in an outer channel to secrete proangiogenic factors and ECM proteins to further induce and stabilize angiogenic sprouts (40). A five-channel device was used by Kim *et al*. (2021) (41) to model angiogenesis in the BBB and determine the role of pericytes or MSCs in creating BBB-like microvasculature. First, lung fibroblasts suspended in a fibrin hydrogel were added to the right outermost channel, and astrocytes suspended in a fibrin gel were added to the center chamber. Then, human BMECs (hBMECs) and human pericytes or human bone marrow MSCs were cultured along the surface of the gel using the left-inner channel. An interstitial flow was induced through the network by increasing the media level in the right-inner channel reservoirs compared to the left-inner channel reservoirs, creating a pressure difference that drives directional flow. The gradient of angiogenic factors released by the lung fibroblasts induced sprouting and invasion of these cell types across the gel. Pericytes were shown to play a role in regulating vessel diameter; however, MSCs also behaved as pericytes by constricting the vessels more efficiently to achieve diameters and vessel architecture closer to that of the BBB. Compared to pericytes, MSCs exhibited a higher expression of PDGFR-β, N-cadherin, and α-SMA and higher secretion of VEGF and SDF-1α. Therefore, MSCs were proposed as a useful substitute for pericytes when modeling the BBB.

Microfluidic platforms of the BBB can also contribute to understanding how pathogens cross the BBB and lead to brain infection. Kim *et al*. (2021) (42) designed a ‘neurovascular-unit-on-a-chip’ to model the BBB penetration of *Cryptococcus neoformans*, a pathogen that causes fungal meningitis. The study used an advanced vertical three-channel device with gravity-driven flow to encapsulate NSCs within a collagen–hyaluronic acid hydrogel and then culture hBMECs and pericytes along the edge of this gel. Two chambers were attached to the channel accessing the EC–pericyte BBB layer where *C. neoformans* was injected. *C. neoformans* appeared to form clusters at the EC–pericyte layer and then penetrate the gel without causing significant changes in the EC tight junctions or continuity of the EC–pericyte layer, suggesting the fungi is transported across the BBB through transcytosis.

## Engineered microvessels

**Figure 2:**
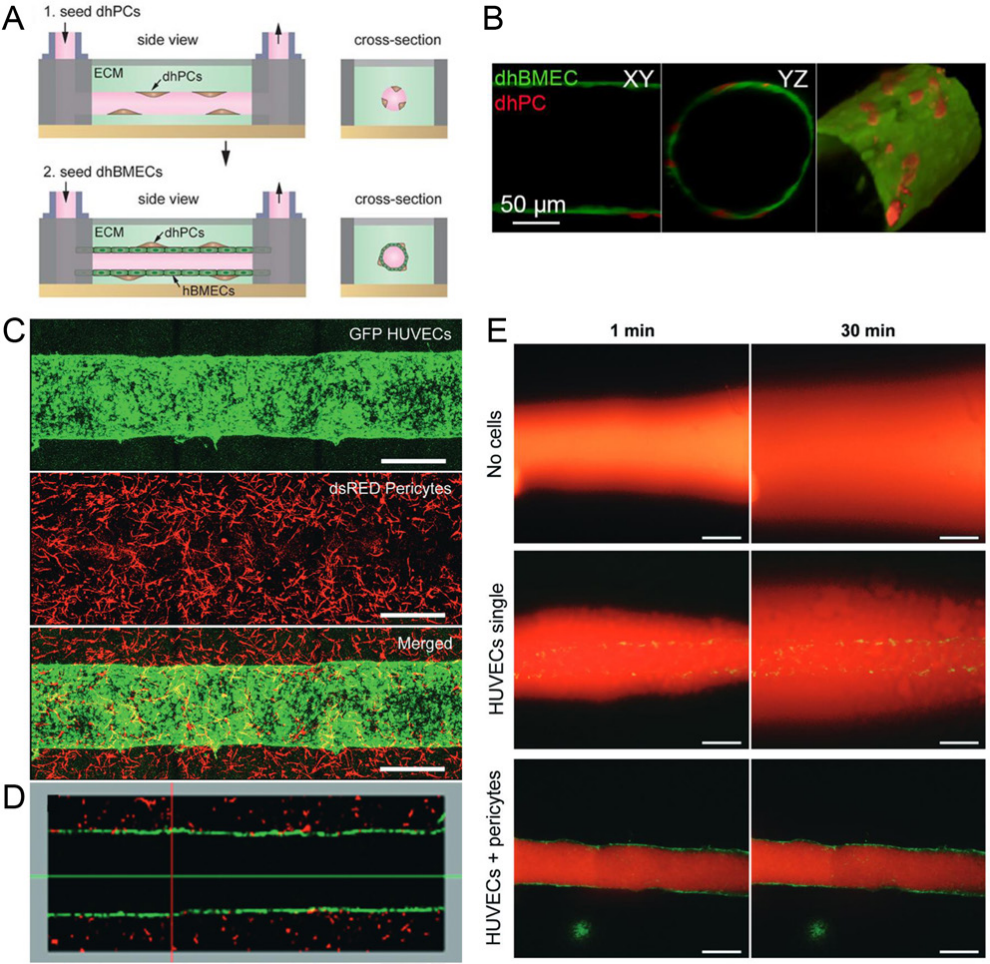
Engineered microvessel platforms. (A) Fabrication of microvessel platform by casting a cylindrical channel in a collagen-I hydrogel and sequentially seeding iPSC-derived pericytes and iPSC-derived BMECs (Jamieson *et al*. (18)). (B) Resulting 3D lumen structure with iPSC-derived BMEC inner monolayer and a discontinuous abluminal layer of iPSC-derived PCs (Jamieson *et al*. (18)). (C) Microvessel platform formed within a fibrinogen hydrogel with an inner monolayer of HUVECs and human brain-derived pericytes suspended in the surrounding matrix. Scale bar = 500 μm (van Dijk *et al*. (46)). (D) Longitudinal cross-section of the engineered microvessel (van Dijk *et al*. (46)). (E) Diffusion of 70 kDa dextran rhodamine 1 min and 30 min after infusion to evaluate barrier function of microvessels with no cells, HUVECs only, and HUVECs and pericytes. Scale bar = 500 μm (van Dijk *et al*. (46)). Arrangement and results were slightly modified for formatting.

Engineered microvessels are biomimetic 3D platforms that seek to recapitulate the luminal structure of blood vessels and serve as a valuable model to study EC–pericyte interactions under fluid flow. Shear stress caused by fluid flow plays a critical role in endothelial function, including migration, proliferation, and gene expression, through mechanosensitive pathways (43). Furthermore, in 2D platforms, fluid flow has been shown to prevent matrix remodeling and maintain vascular quiescence in pericytes in direct contact with ECs, indicating a mechanosensitive response of pericytes to promote vascular stability (44). Vascular permeability is another important function of the vasculature to maintain homeostasis and is partially regulated by EC–pericyte interactions under fluid flow. However, investigating the mechanisms by which pericytes interact with ECs to regulate barrier function has relied on *in vivo* models that lack the ability to external control blood flow and cellular responses. *In vitro* systems incorporating both cell types and allowing for precise fluid flow control through the system could advance basic understanding. Engineered microvessels serve as a useful system to investigate EC–pericyte interactions under shear stress, such as barrier function, through the use of a perfusable lumen. These engineered microvessels are frequently fabricated by injecting a hydrogel, commonly collagen-I, into a PDMS mold where a needle is held in place through the center of the hydrogel chamber (45). After polymerization, the needle is removed, leaving a casted cylindrical channel. ECs and pericytes can be seeded through to channel to form an endothelium lining surrounded by pericytes, and fluid flow can be controlled through access ports. Measuring the permeation of fluorescent-labeled dextran or lucifer yellow (LY) through the endothelial layer is a useful method to quantify barrier function in these systems. For example, Jamieson *et al*. (18) developed a microvessel platform using this method to study the role of iPSC-derived pericytes on the barrier function of iPSC-derived BMECs (Fig. 2A). First, iPSC-derived pericytes were seeded through a channel cast in a collagen-I gel to create a discontinuous outer layer. Then, iPSC-derived BMECs were seeded to form an inner monolayer. As a result, the 3D spatial arrangement of iPSC-derived BMECs surrounded by iPSC-derived pericytes observed *in vivo* was recapitulated (Fig. 2B). Gravity-driven flow was induced through the channel to model physiological shear stress in the postcapillary venules. The permeability of LY through the microvessel was measured, demonstrating that physiologically low barrier function was achieved. No significant differences in LY permeability were measured for the 3D co-culture of iPSC-derived BMECs and pericytes compared to iPSC-derived BMECs alone, indicating that iPSC-derived pericytes did not affect barrier function in this system, despite observing the opposite effect in their 2D models. Contrarily, van Dijk *et al*. (46) showed that human primary brain pericytes positively impacted barrier function in microvessels formed from HUVECs within a fibrinogen gel under continuous, unidirectional flow from a pump system (Fig. 2C, D and E). In this model, human brain pericytes were suspended within the gel solution before polymerization. Then, HUVECs were seeded in the lumen to form an inner monolayer. The human brain pericytes were recruited to the parent vessel over time and assisted ECs in maintaining a monolayer (Fig. 2D). Differences in the role of pericytes on barrier function in these microvessels may result from differences in cell sources, seeding arrangements, shear stress magnitude, and lumen diameter. Recently, Tefft *et al*. (47) used a microvessel model with human brain vascular pericytes and human neonatal dermal blood microvascular ECs to investigate the signaling pathways involved with pericyte stabilization of the endothelium. Pericyte NOTCH3 and EC NOTCH1 played critical roles in forming stable EC junctions. Specifically, pericytes upregulated DLL4 that activated NOTCH1 on ECs to stabilize cell–cell junctions.

Pericytes exist in a quiescent or active state. Pericyte activation is characterized by pericyte detachment and migration, as observed in angiogenesis or tissue regeneration, where pericytes migrate to stabilize developing vasculature. Pericyte activation can also occur in response to inflammation and stress, leading to pericyte loss and aberrant vasculature growth characteristic of numerous disease pathologies, such as diabetic retinopathy (48). However, mechanisms in which pericyte activation is triggered are still widely unknown. Previous studies have revealed engineered microvessels as a useful platform to study the alterations of EC–pericyte interactions in the presence of inflammation signals. For example, Alimperti *et al*. (49) developed a microvessel platform to inform mural cell and EC interactions under exposure to inflammation factors, such as lipopolysaccharide, thrombin, and tumor necrosis factor (TNFα). HUVECs and human bone marrow stromal cells (hBMSCs) were co-cultured in a collagen-I gel and treated with the inflammatory factors. hBMSCs exhibited surface marker expression, abluminal vessel localization, and attachment to the BM similar to mural cells. Furthermore, hBMSCs acted similar to pericytes under inflammation by detaching from the BM and EC monolayer. Specifically, Ras homolog family member A (RhoA) activity increased, leading to less mural cell coverage of the endothelium and increased permeability. The inflammation was also shown to alter Rac1 and N-cadherin, leading to unstable cell–cell junctions and decreased barrier function. Kang *et al*. (50) further investigated the effects of TNF on angiogenic sprouting from the parent vessel by developing a semicylindrical microvessel system in a collagen gel. HUVECs and human primary pericytes were sequentially seeded to form an EC monolayer surrounded by pericytes, and TNFα was diffused through the gel to model inflammation. Low doses of TNFα were shown to enhance angiogenesis; however, high doses of TNFα suppressed angiogenesis. The presence of pericytes appeared to modulate this switch, where the presence of pericytes rescued the antiangiogenic effects of TNFα at high doses to become proangiogenic.

## Organoids

Recently, there has been an increasing interest in miniature 3D organ systems, known as organoids, formed from stem cells *in vitro*. Pluripotent stem cells can self-assemble into 3D organoids, allowing the study of morphogenesis and the role of genetic mutations on cellular function. Blood vessel organoids were established in a seminal study by Wimmer *et al*. (51). Self-assembling human blood vessel organoids composed of ECs and pericytes were formed from PSCs by exposure to various growth factors. To form the organoids, aggregates of human PSCs were differentiated via mesoderm induction and subsequent vascular lineage induction to derive ECs and pericytes (52). Then, the aggregates were encapsulated in a collagen-I hydrogel, where they formed vascular networks and were extracted to create single blood vessel organoids. These vascular networks demonstrated the physiological localization of pericytes surrounding the lumens and the formation of a BM encasing the networks. Single organoids were suspended in individual wells of a low-attachment 96-well plate to allow for high throughput. To study the role of diabetes on EC and pericyte dysfunction, the organoids were exposed to hyperglycemia and inflammatory cytokines. Thickening of the BM was observed, specifically increased deposition of collagen-IV, laminin, and perlecan, resulting from EC and pericyte upregulation of ECM synthesis. Furthermore, splitting of the BM layer was observed, consistent with dermal microvasculature samples from patients with type 2 diabetes. Decreases in the ratio of ECs to pericytes, as well as the absolute cell number for both cell types, were also observed, indicating alterations in EC–pericyte interactions in the diabetic organoids.

Organoids have also been used to model the BBB using ECs, pericytes, and astrocytes to inform the development of brain-penetrating drugs. Simonneau *et al.* (53) developed BBB organoids using human cerebral microvascular ECs, brain microvascular pericytes, and astrocytes to study mechanisms of transport in the BBB. The BBB organoids formed a core of astrocytes surrounded by a layer of pericytes and ECs that acted as a transport barrier. Receptor-mediated antibody transcytosis through the human transferrin receptor was observed. CRISPR/Cas9 was used to investigate the molecular mechanisms governing this transport, which led to the identification of clathrin as a requirement for transferrin receptor-dependent transcytosis in the BBB organoids.

## Discussion/Conclusion

**Figure 3:**
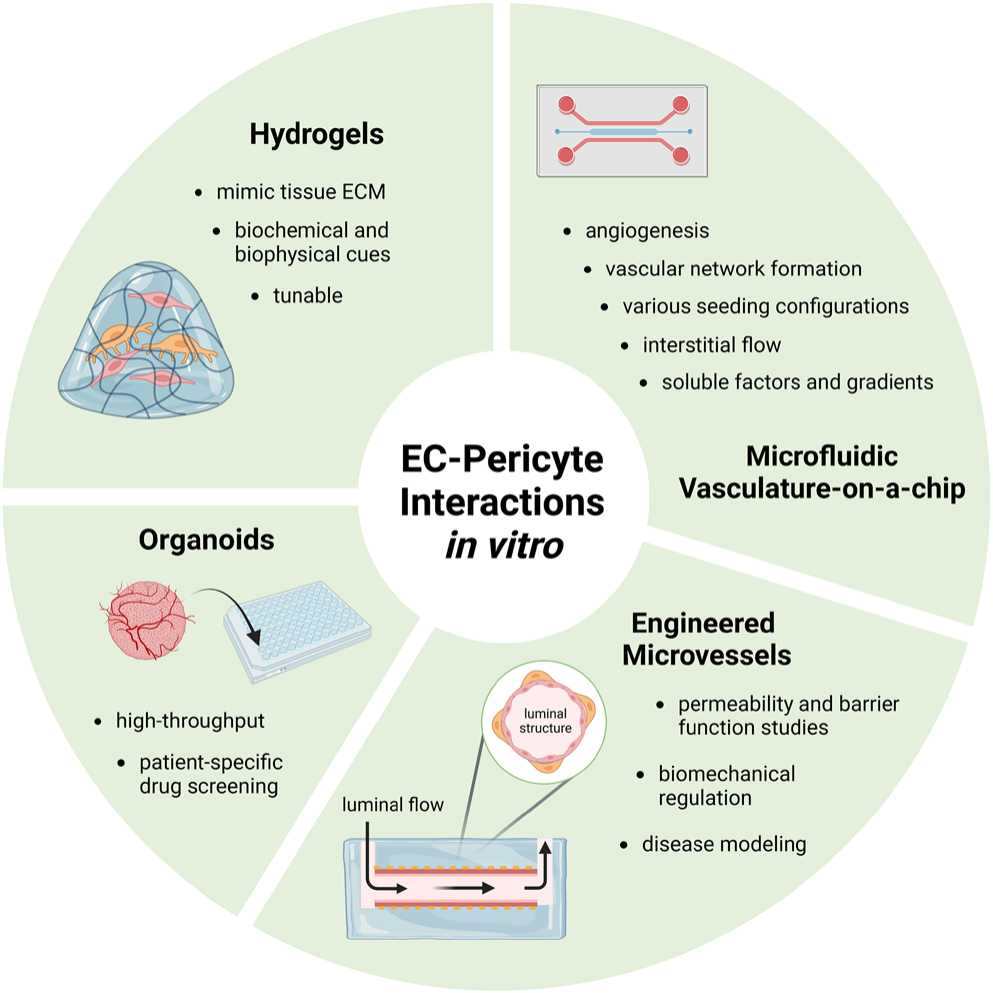
Schematic illustration of *in vitro* systems to model EC–pericyte interactions. Created in BioRender.com

In recent years, significant progress has been made in modeling EC–pericyte interactions *in vitro* to inform vascular development and disease pathologies (Fig. 3). The inherent property of ECs and pericytes to form vascular networks within hydrogels under physiological conditions provides significant opportunities to study their collective role in vascular function, such as signaling pathways involved in pericyte recruitment and localization, barrier function, and BM formation. Utilizing novel microfabrication techniques in combination with hydrogels has advanced these *in vitro* systems by adding directed chemokine gradients, interstitial flow, and complex cell seeding arrangements. Engineered microvessels use hydrogels to recapitulate the 3D cylindrical structure of blood vessels and allow for luminal flow to measure the role of shear stress on these cellular interactions. Altogether, these platforms have contributed to understanding the complex interplay of ECs and pericytes in modulating vascular function. However, there are numerous opportunities for improvement to engineer more physiologically relevant vasculature models with ECs and pericytes *in vitro*.

The microvascular ECM is a complex environment that plays a dynamic role in vascular morphogenesis and guiding vascular cell processes (54). ECM proteins act as signaling entities to ECs and pericytes through integrins to mediate their function. Furthermore, these vascular cells are mechanosensitive and respond to their physical environment. For example, Feng *et al*. (55) demonstrated that hydrogel stiffnesses reminiscent of tumorous tissues induced pericyte–fibroblast transition and increased migration. Thus, the composition and mechanical properties of the hydrogels must be considered when designing physiological *in vitro* systems to model EC–pericyte interactions. Hydrogels derived from ECM proteins, such as collagen-I and Matrigel, are relevant biomaterials due to their natural biochemical cues and biocompatibility. However, these natural biomaterials are characterized by poor mechanical properties and rapid degradation. On the contrary, synthetic hydrogels such as PEG are highly tunable, allowing for modulation of their physical properties and degradation rate, but lack natural biochemical cues. Therefore, there are significant opportunities for designing systems with engineered hydrogels, such as hybrid natural hydrogels with engineered mechanical properties, to mimic the biochemical and biophysical properties ECs and pericytes experience *in vivo*. Furthermore, using engineered hydrogels to elucidate the role of specific microenvironmental cues on EC–pericyte interactions, such as oxygen level or matrix stiffness, is a widely untapped area but will provide insight into numerous disease pathologies characterized by EC–pericyte abnormalities and altered matrix properties. For example, diabetic retinopathy is characterized by pericyte detachment in the microvasculature, leading to decreased blood supply to the retina and hypoxic conditions that exacerbate EC dysfunction. Therefore, hypoxia-inducible hydrogels could be useful for measuring the role of oxygen on altered EC–pericyte function in diabetic retinopathy (56). Furthermore, the interactions between EC and pericytes vary from tissue to tissue, such as the ratio of each cell type (57). Therefore, using tissue-specific cells and considering tissue-specific seeding differences in future *in vitro* systems will help improve model specificity.

Microfluidic and engineered microvessel platforms have gained significant popularity as co-culture systems for ECs and pericytes. However, these platforms pose numerous challenges, such as proper cell seeding and even cell distribution, which can lead to variability in results. Standardizing the seeding protocols for these devices will contribute to eliminating these variabilities. However, exploring different seeding configurations and spatial arrangements is an advantage of these devices that may require more work to standardize. An additional limitation in using microvessel platforms to model EC–pericyte interactions is the relatively large diameter of these vessels. The microvessel platforms reviewed here range from 160 to 500 μm in diameter; however, capillaries are only 1–10 μm in diameter. Therefore, these ‘micro’vessel platforms should be discussed more in the context of larger vessels, such as arteries/arterioles and veins/venules, rather than the microvasculature. Furthermore, pericytes are not present on larger diameter vessels; therefore, the relevance of these platforms to study EC–pericyte interactions is drawn into question. Zhao *et al*. (58) recently developed a novel microvessel platform with capillary-scale lumens, but these vessels lacked pericytes due to challenges in creating perfusable lumens with multiple cell types. Therefore, constructing microvessel platforms with both cell types and diameters closer in size to the capillaries warrants further attention. As mentioned, microfluidic devices are commonly made using PDMS due to its ease of use, biocompatibility, and gas permeability. However, PDMS also leads to drug absorption, which presents challenges during drug studies (59). This challenge can be overcome by using mass spectroscopy to account for the effects of PDMS, but it adds complexity to using these platforms for drug studies. Organoids are also a promising platform for drug studies for diseases impacting EC–pericyte interactions as they allow for high-throughput screening, and patient-derived cells can be used for patient-specific screens.

Overall, the *in vitro* systems reviewed here have proven useful platforms to inform the vasculature's complex relationship between ECs and pericytes. However, numerous questions still remain or have yet to be addressed with regard to the physiological relevance of these platforms. The verification of pericyte function in these systems is often limited to immunolabeling and comparative effects on permeability. It remains unclear whether pericyte function as observed *in vivo* is really mimicked in these platforms; therefore, researchers must consider the degree of pericyte function in these systems to understand how they can be used to inform EC–pericyte interactions. Further discussion and improvements in these systems will promote a deeper understanding of these interactions *in vivo* and continue to advance the field of tissue engineering.

## Declaration of interest

The authors declare that there is no conflict of interest that could be perceived as prejudicing the impartiality of the research reported.

## Funding

This work did not receive any specific grant from any funding agency in the public, commercial, or not-for-profit sector.

## References

[1] Jamieson J Macklin B Gerecht S. Pericytes derived from human pluripotent stem cells. Advances in Experimental Medicine and Biology 2018 1109 111–124. (10.1007/978-3-030-02601-1_9) 30523593

[2] Wanjare M Kusuma S Gerecht S. Defining differences among perivascular cells derived from human pluripotent stem cells. Stem Cell Reports 2014 2 561–575. (10.1016/j.stemcr.2014.03.004) 24936446 PMC4050491

[3] Armulik A Genové G Betsholtz C. Pericytes: developmental, physiological, and pathological perspectives, problems, and promises. Developmental Cell 2011 21 193–215. (10.1016/j.devcel.2011.07.001) 21839917

[4] Dore-Duffy P Cleary K. Morphology and properties of pericytes. In Nag SEd. The Blood-Brain and Other Neural Barriers [Internet]. Totowa, NJ: Humana Press, pp. 49–68. 2011. [cited 2022 Nov 7]. Available at: http://link.springer.com/10.1007/978-1-60761-938-3_2 10.1007/978-1-60761-938-3_2 21082366

[5] Bergers G Song S. The role of pericytes in blood-vessel formation and maintenance. Neuro-Oncology 2005 7 452–464. (10.1215/S1152851705000232) 16212810 PMC1871727

[6] Sengillo JD Winkler EA Walker CT Sullivan JS Johnson M Zlokovic BV. Deficiency in mural vascular cells coincides with blood-brain barrier disruption in Alzheimer’s disease. Brain Pathology 2013 23 303–310. (10.1111/bpa.12004) 23126372 PMC3628957

[7] Hammes HP Lin J Renner O Shani M Lundqvist A Betsholtz C Brownlee M Deutsch U. Pericytes and the pathogenesis of diabetic retinopathy. Diabetes 2002 51 3107–3112. (10.2337/diabetes.51.10.3107) 12351455

[8] Morikawa S Baluk P Kaidoh T Haskell A Jain RK McDonald DM. Abnormalities in pericytes on blood vessels and endothelial sprouts in tumors. American Journal of Pathology 2002 160 985–1000. (10.1016/S0002-9440(1064920-6) 11891196 PMC1867175

[9] Gaengel K Genové G Armulik A Betsholtz C. Endothelial-mural cell signaling in vascular development and angiogenesis. Arteriosclerosis, Thrombosis, and Vascular Biology 2009 29 630–638. (10.1161/ATVBAHA.107.161521) 19164813

[10] Lindahl P Johansson BR Levéen P Betsholtz C. Pericyte loss and microaneurysm formation in PDGF-B-deficient mice. Science 1997 277 242–245. (10.1126/science.277.5323.242) 9211853

[11] Augustin HG Young Koh GY Thurston G Alitalo K. Control of vascular morphogenesis and homeostasis through the angiopoietin–Tie system. Nature Reviews. Molecular Cell Biology 2009 10 165–177. (10.1038/nrm2639) 19234476

[12] Sweeney MD Ayyadurai S Zlokovic BV. Pericytes of the neurovascular unit: key functions and signaling pathways. Nature Neuroscience 2016 19 771–783. (10.1038/nn.4288) 27227366 PMC5745011

[13] Payne LB Tewari BP Dunkenberger L Bond S Savelli A Darden J Zhao H Willi C Kanodia R Gude R Pericyte progenitor coupling to the emerging endothelium during vasculogenesis via connexin 43. Arteriosclerosis, Thrombosis, and Vascular Biology 2022 42. e96–e114. (10.1161/ATVBAHA.121.317324) 35139658 PMC8957572

[14] Kruse K Lee QS Sun Y Klomp J Yang X Huang F Sun MY Zhao S Hong Z Vogel SM N-cadherin signaling via Trio assembles adherens junctions to restrict endothelial permeability. Journal of Cell Biology 2019 218 299–316. (10.1083/jcb.201802076) 30463880 PMC6314553

[15] Blatchley MR Hall F Wang S Pruitt HC Gerecht S. Hypoxia and matrix viscoelasticity sequentially regulate endothelial progenitor cluster-based vasculogenesis. Science Advances 2019 5 eaau7518. (10.1126/sciadv.aau7518) PMC6426463 30906859

[16] Wei Z Schnellmann R Pruitt HC Gerecht S. Hydrogel network dynamics regulate vascular morphogenesis. Cell Stem Cell 2020 27 798–812.e6. (10.1016/j.stem.2020.08.005) 32931729 PMC7655724

[17] Schnellmann R Ntekoumes D Choudhury MI Sun S Wei Z Gerecht S. Stiffening matrix induces age‐mediated microvascular phenotype through increased cell contractility and destabilization of adherens junctions. Advanced Science 2022 9 e2201483. (10.1002/advs.202201483) 35657074 PMC9353494

[18] Jamieson JJ Linville RM Ding YY Gerecht S Searson PC. Role of iPSC-derived pericytes on barrier function of iPSC-derived brain microvascular endothelial cells in 2D and 3D. Fluids and Barriers of the CNS 2019 16 15. (10.1186/s12987-019-0136-7) PMC6551886 31167667

[19] Mannino G Gennuso F Giurdanella G Conti F Drago F Salomone S Furno DL Bucolo C Giuffrida R. Pericyte-like differentiation of human adipose-derived mesenchymal stem cells: an *in vitro* study. World Journal of Stem Cells 2020 12 1152–1170. (10.4252/wjsc.v12.i10.1152) 33178398 PMC7596446

[20] Kato K Diéguez-Hurtado R Park DY Hong SP Kato-Azuma S Adams S Stehling M Trappmann B Wrana JL Koh GY Pulmonary pericytes regulate lung morphogenesis. Nature Communications 2018 9 2448. (10.1038/s41467-018-04913-2) PMC6015030 29934496

[21] Antoine EE Vlachos PP Rylander MN. Review of collagen I hydrogels for bioengineered tissue microenvironments: characterization of mechanics, structure, and transport. Tissue Engineering. Part B, Reviews 2014 20 683–696. (10.1089/ten.TEB.2014.0086) 24923709 PMC4241868

[22] Kemp SS Aguera KN Cha B Davis GE. Defining endothelial cell-derived factors that promote pericyte recruitment and capillary network assembly. Arteriosclerosis, Thrombosis, and Vascular Biology 2020 40 2632–2648. (10.1161/ATVBAHA.120.314948) 32814441 PMC7939110

[23] Bowers SLK Kemp SS Aguera KN Koller GM Forgy JC Davis GE. Defining an upstream VEGF (vascular endothelial growth factor) priming signature for downstream factor-induced endothelial cell-pericyte tube network coassembly. Arteriosclerosis, Thrombosis, and Vascular Biology 2020 40 2891–2909. (10.1161/ATVBAHA.120.314517) 33086871 PMC7939123

[24] Stratman AN Schwindt AE Malotte KM Davis GE. Endothelial-derived PDGF-BB and HB-EGF coordinately regulate pericyte recruitment during vasculogenic tube assembly and stabilization. Blood 2010 116 4720–4730. (10.1182/blood-2010-05-286872) 20739660 PMC2996127

[25] Yamazaki Y Shinohara M Yamazaki A Ren Y Asmann YW Kanekiyo T Bu G. ApoE (apolipoprotein E) in brain pericytes regulates endothelial function in an isoform-dependent manner by modulating basement membrane components. Arteriosclerosis, Thrombosis, and Vascular Biology 2020 40 128–144. (10.1161/ATVBAHA.119.313169) 31665905 PMC7007705

[26] Kleinman HK Martin GR. Matrigel: basement membrane matrix with biological activity. Seminars in Cancer Biology 2005 15 378–386. (10.1016/j.semcancer.2005.05.004) 15975825

[27] Blanchard JW Bula M Davila-Velderrain J Akay LA Zhu L Frank A Victor MB Bonner JM Mathys H Lin YT Reconstruction of the human blood–brain barrier in vitro reveals a pathogenic mechanism of APOE4 in pericytes. Nature Medicine 2020 26 952–963. (10.1038/s41591-020-0886-4) PMC7704032 32514169

[28] Jack CR Knopman DS Jagust WJ Petersen RC Weiner MW Aisen PS Shaw LM Vemuri P Wiste HJ Weigand SD Tracking pathophysiological processes in Alzheimer’s disease: an updated hypothetical model of dynamic biomarkers. Lancet. Neurology 2013 12 207–216. (10.1016/S1474-4422(1270291-0) 23332364 PMC3622225

[29] Vukicevic S Kleinman HK Luyten FP Roberts AB Roche NS Reddi AH. Identification of multiple active growth factors in basement membrane matrigel suggests caution in interpretation of cellular activity related to extracellular matrix components. Experimental Cell Research 1992 202 1–8. (10.1016/0014-4827(9290397-q) 1511725

[30] Desai BJ Gruber HE Hanley EN. The influence of Matrigel or growth factor reduced Matrigel on human intervertebral disc cell growth and proliferation. Histology and Histopathology 1999 14 359–368. (10.14670/HH-14.359) 10212797

[31] Matta R Yousafzai MS Murrell M Gonzalez AL. Endothelial cell secreted metalloproteinase-2 enhances neural stem cell N-cadherin expression, clustering, and migration. FASEB Journal 2021 35 e21311. (10.1096/fj.202002302RR) 33417253

[32] Roudsari LC Jeffs SE Witt AS Gill BJ West JL. A 3D poly(ethylene glycol)-based Tumor angiogenesis Model to Study the Influence of Vascular Cells on Lung Tumor Cell Behavior. Scientific Reports 2016 6 32726. (10.1038/srep32726) PMC5011743 27596933

[33] Ewald ML Chen YH Lee AP Hughes CCW. The vascular niche in next generation microphysiological systems. Lab on a Chip 2021 21 3244–3262. (10.1039/d1lc00530h) 34396383 PMC8635227

[34] Wan Z Zhong AX Zhang S Pavlou G Coughlin MF Shelton SE Nguyen HT Lorch JH Barbie DA Kamm RD. A robust method for perfusable microvascular network formation in vitro. Small Methods 2022 6 e2200143. (10.1002/smtd.202200143) 35373502 PMC9844969

[35] Sia SK Whitesides GM. Microfluidic devices fabricated in poly(dimethylsiloxane) for biological studies. Electrophoresis 2003 24 3563–3576. (10.1002/elps.200305584) 14613181

[36] Bai J Khajavi M Sui L Fu H Tarakkad Krishnaji S Birsner AE Bazinet L Kamm RD D’Amato RJ. Angiogenic responses in a 3D micro-engineered environment of primary endothelial cells and pericytes. Angiogenesis 2021 24 111–127. (10.1007/s10456-020-09746-6) 32955682

[37] Uwamori H Ono Y Yamashita T Arai K Sudo R. Comparison of organ-specific endothelial cells in terms of microvascular formation and endothelial barrier functions. Microvascular Research 2019 122 60–70. (10.1016/j.mvr.2018.11.007) 30472038 PMC6294313

[38] Yamamoto K Tanimura K Watanabe M Sano H Uwamori H Mabuchi Y Matsuzaki Y Chung S Kamm RD Tanishita K Construction of continuous capillary networks stabilized by pericyte-like perivascular cells. Tissue Engineering. Part A 2019 25 499–510. (10.1089/ten.TEA.2018.0186) 30234439

[39] Campisi M Shin Y Osaki T Hajal C Chiono V Kamm RD. 3D self-organized microvascular model of the human blood-brain barrier with endothelial cells, pericytes and astrocytes. Biomaterials 2018 180 117–129. (10.1016/j.biomaterials.2018.07.014) 30032046 PMC6201194

[40] Nakatsu MN Sainson RCA Aoto JN Taylor KL Aitkenhead M Pérez-del-Pulgar S Carpenter PM Hughes CC. Angiogenic sprouting and capillary lumen formation modeled by human umbilical vein endothelial cells (HUVEC) in fibrin gels: The role of fibroblasts and Angiopoietin-1. Microvascular Research 2003 66 102–112. (10.1016/s0026-2862(0300045-1) 12935768

[41] Kim S Lee S Lim J Choi H Kang H Jeon NL Son Y. Human bone marrow-derived mesenchymal stem cells play a role as a vascular pericyte in the reconstruction of human BBB on the angiogenesis microfluidic chip. Biomaterials 2021 279 121210. (10.1016/j.biomaterials.2021.121210) 34710793

[42] Kim J Lee KT Lee JS Shin J Cui B Yang K Choi YS Choi N Lee SH Lee JH Fungal brain infection modelled in a human-neurovascular-unit-on-a-chip with a functional blood–brain barrier. Nature Biomedical Engineering 2021 5 830–846. (10.1038/s41551-021-00743-8) 34127820

[43] Li YSJ Haga JH Chien S. Molecular basis of the effects of shear stress on vascular endothelial cells. Journal of Biomechanics 2005 38 1949–1971. (10.1016/j.jbiomech.2004.09.030) 16084198

[44] Schrimpf C Koppen T Duffield JS Böer U David S Ziegler W Haverich A Teebken OE Wilhelmi M. TIMP3 is regulated by pericytes upon shear stress detection leading to a modified endothelial cell response. European Journal of Vascular and Endovascular Surgery 2017 54 524–533. (10.1016/j.ejvs.2017.07.002) 28807411

[45] Polacheck WJ Kutys ML Tefft JB Chen CS. Microfabricated blood vessels for modeling the vascular transport barrier. Nature Protocols 2019 14 1425–1454. (10.1038/s41596-019-0144-8) 30953042 PMC7046311

[46] van Dijk CGM Brandt MM Poulis N Anten J van der Moolen M Kramer L Homburg EFGA Louzao-Martinez L Pei J Krebber MM A new microfluidic model that allows monitoring of complex vascular structures and cell interactions in a 3D biological matrix. Lab on a Chip 2020 20 1827–1844. (10.1039/d0lc00059k) 32330215

[47] Tefft JB Bays JL Lammers A Kim S Eyckmans J Chen CS. Notch1 and notch3 coordinate for pericyte-induced stabilization of vasculature. American Journal of Physiology-Cell Physiology 2022 322 C185–C196. (10.1152/ajpcell.00320.2021) 34878922 PMC8791789

[48] To M Goz A Camenzind L Oertle P Candiello J Sullivan M Henrich PB Loparic M Safi F Eller A Diabetes-induced morphological, biomechanical, and compositional changes in ocular basement membranes. Experimental Eye Research 2013 116 298–307. (10.1016/j.exer.2013.09.011) 24095823

[49] Alimperti S Mirabella T Bajaj V Polacheck W Pirone DM Duffield J Eyckmans J Assoian RK Chen CS. Three-dimensional biomimetic vascular model reveals a RhoA, Rac1, and *N* -cadherin balance in mural cell–endothelial cell-regulated barrier function. Proceedings of the National Academy of Sciences of the United States of America 2017 114 8758–8763. (10.1073/pnas.1618333114) 28765370 PMC5565405

[50] Kang TY Bocci F Jolly MK Levine H Onuchic JN Levchenko A. Pericytes enable effective angiogenesis in the presence of proinflammatory signals. Proceedings of the National Academy of Sciences of the United States of America 2019 116 23551–23561. (10.1073/pnas.1913373116) 31685607 PMC6876202

[51] Wimmer RA Leopoldi A Aichinger M Wick N Hantusch B Novatchkova M Taubenschmid J Hämmerle M Esk C Bagley JA Human blood vessel organoids as a model of diabetic vasculopathy. Nature 2019 565 505–510. (10.1038/s41586-018-0858-8) 30651639 PMC7116578

[52] Wimmer RA Leopoldi A Aichinger M Kerjaschki D Penninger JM. Generation of blood vessel organoids from human pluripotent stem cells. Nature Protocols 2019 14 3082–3100. (10.1038/s41596-019-0213-z) 31554955

[53] Simonneau C Duschmalé M Gavrilov A Brandenberg N Hoehnel S Ceroni C Lassalle E Kassianidou E Knoetgen H Niewoehner J Investigating receptor-mediated antibody transcytosis using blood–brain barrier organoid arrays. Fluids and Barriers of the CNS 2021 18 43. (10.1186/s12987-021-00276-x) PMC8454074 34544422

[54] Davis GE Senger DR. Endothelial extracellular matrix: biosynthesis, remodeling, and functions during vascular morphogenesis and neovessel stabilization. Circulation Research 2005 97 1093–1107. (10.1161/01.RES.0000191547.64391.e3) 16306453

[55] Feng F Feng X Zhang D Li Q Yao L. Matrix stiffness induces pericyte-fibroblast transition through YAP activation. Frontiers in Pharmacology 2021 12 698275. (10.3389/fphar.2021.698275) PMC8202079 34135765

[56] Chan XY Black R Dickerman K Federico J Lévesque M Mumm J Gerecht S. Three-dimensional vascular network assembly from diabetic patient-derived induced pluripotent stem cells. Arteriosclerosis, Thrombosis, and Vascular Biology 2015 35 2677–2685. (10.1161/ATVBAHA.115.306362) 26449749 PMC4603427

[57] Armulik A Abramsson A Betsholtz C. Endothelial/pericyte interactions. Circulation Research 2005 97 512–523. (10.1161/01.RES.0000182903.16652.d7) 16166562

[58] Zhao N Guo Z Kulkarni S Norman D Zhang S Chung TD Nerenberg RF Linville R Searson P. Engineering the human blood–brain barrier at the capillary scale using a double‐templating technique. Advanced Functional Materials 2022 32 2110289. (10.1002/adfm.202110289) PMC9610437 36312050

[59] Ingber DE Human organs-on-chips for disease modelling, drug development and personalized medicine. Nature Reviews. Genetics 2022 23 467–491. (10.1038/s41576-022-00466-9) PMC8951665 35338360

